# A blinded randomised controlled trial to determine the effect of enteric coating on enzyme treatment for canine exocrine pancreatic efficiency

**DOI:** 10.1186/1746-6148-8-127

**Published:** 2012-07-28

**Authors:** Aran Mas, Peter-John M Noble, Peter J Cripps, Daniel J Batchelor, Peter Graham, Alexander J German

**Affiliations:** 1 School of Veterinary Science, University of Liverpool, Neston, United Kingdom; 2 Nationwide Laboratories Lancefield House, 23 Mains Lane, Poulton-le-Fylde, United Kingdom

**Keywords:** Dog, Pancreas, Malabsorption, Diarrhoea, Lipase, Trypsin

## Abstract

**Background:**

Enzyme treatment is the mainstay for management of exocrine pancreatic insufficiency (EPI) in dogs. ‘Enteric-coated’ preparations have been developed to protect the enzyme from degradation in the stomach, but their efficacy has not been critically evaluated. The hypothesis of the current study was that enteric coating would have no effect on the efficacy of pancreatic enzyme treatment for dogs with EPI.

Thirty-eight client-owned dogs with naturally occurring EPI were included in this multicentre, blinded, randomised controlled trial. Dogs received either an enteric-coated enzyme preparation (test treatment) or an identical preparation without the enteric coating (control treatment) over a period of 56 days.

**Results:**

There were no significant differences in either signalment or cobalamin status (where cobalamin deficient or not) between the dogs on the test and control treatments. Body weight and body condition score increased in both groups during the trial (*P*<0.001) but the magnitude of increase was greater for the test treatment compared with the control treatment (*P*<0.001). By day 56, mean body weight increase was 17% (95% confidence interval 11-23%) in the test treatment group and 9% (95% confidence interval 4-15%) in the control treatment group. The dose of enzyme required increased over time (*P*<0.001) but there was no significant difference between treatments at any time point (*P*=0.225). Clinical disease severity score decreased over time for both groups (*P*=0.011) and no difference was noted between groups (*P*=0.869). No significant adverse effects were reported, for either treatment, for the duration of the trial.

**Conclusions:**

Enteric coating a pancreatic enzyme treatment improves response in canine EPI.

## Background

Exocrine pancreatic insufficiency (EPI) is a common condition in dogs, resulting from inadequate functional reserve of pancreatic acinar tissue [1]. The most common cause of EPI is pancreatic acinar atrophy, although other causes have been reported, including chronic pancreatitis, pancreatic neoplasia and (possibly) congenital hypoplasia. Clinical signs only develop when a critical mass (e.g. >90%) of exocrine tissue has been lost, and result from maldigestion and subsequent malabsorption.

Clinical management usually involves enzyme replacement therapy with the addition of dietary modification (e.g. highly digestible diet) and ancillary therapies (e.g. antibacterials) if response to enzyme alone is poor [1]. Most dried pancreatic extracts are given as a powdered formulation, although ‘enteric-coated’ preparations have been developed in which granules of enzyme powder are coated in a lacquer that protects the enzymes from degradation in the stomach. However, whilst widely used, the effectiveness of enteric-coated preparation has been questioned, and one study found that such formulations were less effective than uncoated preparations [2]. In contrast, a more recent study found no difference in response or long term survival between those dogs taking uncoated and coated preparations [3]. All studies to date have suffered from the limitation that they are retrospective and uncontrolled. Thus, the true effect that enteric coating has on efficacy remains unclear. As a result, there is a need to determine whether a difference in efficacy exists between types of enzyme supplementation used for the treatment of canine EPI. Given the conflicting information from previous studies, our chosen hypothesis was that enteric coating of a pancreatic enzyme extract would have no effect on the efficacy of treatment for canine EPI. Our aim was to conduct the first blinded randomised controlled trial (RCT) assessing therapeutic efficacy in this condition.

## Methods

### Trial design and objectives

The study was a multicentre randomised blinded ‘positive controlled’ trial, and used a two-group parallel design. The main objective was to determine the effect of enteric coating on efficacy of a pancreatic enzyme supplement in the treatment of canine EPI. The studied complied with the University of Liverpool Guidelines on Animal Welfare and Experimentation, and was approved by the University of Liverpool Research Ethics Committee (RETH000328). Prior to enrolment, owners were informed as to the nature of the study and gave their informed consent in writing. At the end of the trial, all owners were asked to complete a trial feedback form to ensure that they were happy with trial conduct. As far as possible (for a trial in a veterinary species), the studied complied with the principles of Good Clinical Practice (GCP) [4].

### Study subjects

Cases were recruited between March 2009 and July 2011, when the target for enrolment had been reached. Dogs recently (within two weeks) diagnosed with EPI (i.e. serum trypsin-like immunoreactivity [TLI] less than the lower limit of the respective laboratory reference interval, typically 2.0-3.0 μg/L) were eligible for enrolment. Other eligibility criteria included absence of any concurrent disease that might affect diagnosis, response to treatment (especially body weight gain) or prognosis including concurrent cardiac disease, renal disease, hepatic disease, endocrine disease or neoplasia. Further, it was a requirement that routine haematological and serum biochemical analysis had been performed within 4 weeks of enrolment. Finally, cases could not have previously been treated with pancreatic enzyme supplementation.

Any first-opinion veterinary practice with a case fitting the inclusion criteria was eligible to request enrolment in the trial. When a request was made, one of the study observers (AM, PJN, AJG) discussed the study outline with the primary care veterinarian, who in turn discussed the trial with the owner of the eligible dog. Assuming that the practice remained interested, the owner and primary care veterinarian could decide whether or not the trial visits were conducted at the Small Animal Teaching Hospital (SATH), or at the practice of their primary care veterinarian. If owners and/or veterinarians preferred the latter, a detailed study pack was then posted (by next-day recorded delivery). This pack contained a detailed information brochure for the veterinarian, explaining their duties as a trial investigator, an owner information sheet, a consent form, a form to record details of all visits and client communication, and a 9-point body condition score (BCS) chart [5]. In addition, the specific therapy, for the whole of the trial, was also provided (see below). For cases that were enrolled at the SATH, the owner information sheet and consent form were posted to the client prior to the first appointment.

### Trial publicity and incentives

In order to maximise recruitment, the trial was advertised in a number of ways, including mailshots to local veterinary practices, advertising footnotes to referral letters (written by all clinicians within the SATH for the duration of the trial), letters submitted to the veterinary press (e.g. Veterinary Record), advertising by the trial sponsor when their company representatives visited first opinion practices, and sponsored continuing education meetings organised for first-opinion veterinarians. In addition, a footnote advertising the trial was added to the results report for any TLI test result diagnostic for EPI at a large commercial clinical pathology laboratory (NationWide Laboratories, Poulton-le-Fylde, UK). In all cases, veterinarians with potentially eligible cases were encouraged to contact the study observers for details.

For cases where the owner wished to attend the SATH for their appointments, the costs of travel were reimbursed. In addition, the study medication was provided free of charge. Finally, to encourage compliance, any costs for the contributing primary veterinarians were defrayed by a payment of £300 for every case that completed the trial where all study paperwork was returned in a timely manner. Study observers did not receive any incentives or remuneration for completing the trial.

### Roles and responsibilities

For cases seen at the SATH, the study observers (AM, PJN, AJG) were responsible for liaising with the owners, performing the examination at each visit and modifying therapy when necessary. The respective primary care veterinarian was informed of case progress throughout by letter. In the cases not referred to SATH, the primary care veterinarian was responsible for examining and managing the dog, for liaising with the client, and for completing study paperwork. However, they could contact the study observers at any time if they had questions regarding case management.

### Treatments

Both the test treatment and control treatment were based upon a commercially-available porcine pancreatic enzyme extract with an enteric coating, designed to protect the active enzyme from acid digestion and ensure high concentrations reach the small intestine (Lypex, Vet Plus Ltd., Lytham, UK; 30,000 ph Eur U lipase, 18750 ph Eur U amylase, 1200 ph Eur U protease per capsule). This product is based on Pancreatin, a highly active, porcine-derived enzyme combination. After active ingredient extraction, the dried enzyme is then pelleted and contains no excipient. An enteric coating is then applied, which is added in a solvent-free polymethacrylic acid/ester process using a dispersion film former. The coated pellets range from 1.4-2.4 mm in size. The product was manufactured by Nordmark Arzneimittel GmbH & Co. KG (Uetersen, Germany), on behalf of VetPlus Ltd.

The test treatment was identical to the commercially available product; the control treatment was similar in all aspects, except that it lacked the enteric coating, and was not commercially available. However, the organoleptic properties were identical and both treatments were presented in similar plain packaging (see below), ensuring that test and control treatment could not be distinguished. Nordmark Arzneimittel purpose-formulated both treatments for the trial on 24/11/08, with an expiry date of 30/11/11. Efficacy was tested and confirmed and the product was certified to be free from microbial contamination. Sufficient treatment was manufactured for a total of 40 dogs (with 20 receiving the test treatment, and 20 receiving the control product) and, in order to budget for difference in dogs size, enough product was manufactured to last 2 months even for a large dog where dose increases were required at each visit (see below).

The same starting dose of enzyme was used for both treatments: one capsule per day was administered to dogs <10 kg, divided over two meals, whilst 2 capsules/day (1 capsule per meal) was given to dogs >10 kg. During administration, the gelatin capsule was opened and the product mixed well with the food immediately prior to feeding. In order to avoid the risk of any skin irritation, owners were instructed to wear gloves when handling the capsules.

### Initial assessment and enrolment

During the initial assessment, a detailed medical history was taken, physical examination performed, body weight was measured, and body condition was scored [5]. Information about the severity of clinical signs was then obtained from the owner, using a standardised system (Table 1). This enabled each clinical sign (i.e. appetite, frequency of defecation, faecal consistency, vomiting, flatulence, borborygmus, coprophagia and attitude/activity), to be scored semi-quantitatively, in a manner similar to another clinical scoring system used for chronic enteropathy [6].

**Table 1:** The clinical signs scoring system used two assess efficacy of two enzyme treatments for canine exocrine pancreatic insufficiency

**Criterion**	**Description**	**Score**
Attitude/activity	Dull, unwilling to exercise	3
	Decreased activity	1
	Normal activity	0
	Increased activity	0
Appetite	Poor	3
	Normal	0
	Good	1
	Excessive	3
Vomiting	None	0
	1-2/week	1
	3-4/week	2
	≥5/week	3
Defecation frequency	1-2/day	0
	3-4/day	1
	5-6/day	2
	≥7/day	3
Fecal consistency	Normal	0
	Moist and poorly formed	1
	Pulp-like	2
	Watery diarrhoea	3
Flatulence	None	0
	Occasional	1
	Often	3
Borborygmus	None	0
	Occasional	1
	Often	3
Coprophagia	Present	3
	Absent	0

Clinical signs of dogs with EPI were assessed at each study visit. All clinical signs listed above were assigned a score from 0 to 3, as in the table. Individual clinical signs scores were then added together to generate a global score, with a minimum of 0 (no signs) and a maximum of 24 (severe signs).

At this stage, the purpose of the trial was again discussed with the owner and, assuming that they were happy, they were asked to sign the study consent form. Thereafter, dogs were allocated a study number and the treatment dispensed. The owners were instructed on how to use the treatment and any specific questions that the owner had were answered at this stage.

### Monitoring, treatment alterations and follow-up

**Figure 1:**
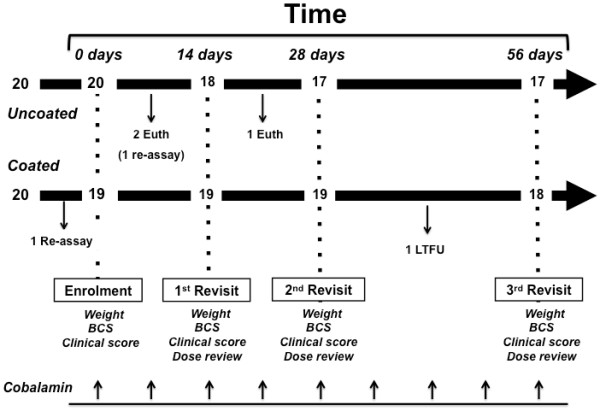
**Summary of the trial design and inclusion of dogs.** Forty dogs, with exocrine pancreatic insufficiency, were randomised to receive one of two enzyme treatments (test e.g. coated and control e.g. uncoated), and were re-examined at 3 follow-up visits (day 14, day 28 and day 56). Information in the lower part of the figure refers to the procedures conducted at each visit. BCS: body condition score; LTFU: lost to follow up. Arrows indicate when doses of parenteral cobalamin were injected. Other than the timeline, all numbers refer to dogs recruited and remaining within the trial at the respective points.

A summary of the study protocol is given in Figure 1. Throughout the trial, dogs returned on a weekly basis for administration of subcutaneous cobalamin (see below). Detailed assessments were conducted on days 14, 28 and 56. At each visit, a physical examination was performed, body weight was measured (using the same electronic scales as for the first visit), and a body condition score was performed. In addition, clinical signs were again scored using the same questionnaire as for the initial visit. Compliance with administration of the treatment was confirmed and, if necessary, dosage alterations were made and additional therapy was added.

In addition to the official re-evaluation visits, clients contacted either the attending veterinarian or study observers using telephone calls and, occasionally, e-mail updates.

### Treatment alterations, use of additional therapy, and diet

The intention was that only the enzyme supplement and weekly cobalamin injections (see below) would be administered during the trial. However, in accordance with normal clinical practice, alterations in enzyme dose and the addition of other therapies were allowed if response to therapy was poor. Primary care veterinarians made all treatment changes, after discussing them with one of the study observers, and in accordance with a standardised protocol. In this regard if, at the first reassessment (14 days), clinical response was deemed to be insufficient (e.g. poor weight gain, lack of resolution of clinical signs), the dose of enzyme was doubled. A further enzyme dosage increases were allowed on days 28 and 56, if necessary.

Given that hypocobalaminaemia is a negative prognostic indicator in canine EPI [3], and in order to ensure that this was not a confounding factor during the trial, weekly subcutaneous injections of cobalamin (at 20 μg/kg) were administered to all dogs. This treatment was given irrespective of whether hypocobalaminaemia was present in pre-trial serum biochemical results. No other treatment was initially allowed in any of the dogs. Additional therapies were avoided as far as possible but if, in the opinion of the attending veterinarian they were deemed to be necessary, then they could be added from third visit (week 4) onwards, again after discussion with the study observers. Sanctioned additional therapies included the use of either antibacterials (e.g. oxytetracycline at 10 mg/kg q8 h PO, or metronidazole at 10 mg/kg q12 h PO) or histamine-2-receptor antagonists (e.g. ranitidine at 2 mg/kg q12h PO).

In order to avoid any potential cofounding effect of a diet change on treatment response, all owners were instructed to continuing feeding their dog’s existing diet at the same level. In all cases, dogs were fed twice daily. Details of exact diets fed were not recorded.

### Patient welfare, adverse events, early trial discontinuation, and euthanasia

Throughout the study, all efforts were made to safeguard the welfare of the dogs enrolled, and owners were free to withdraw at any stage. The attending veterinarian recorded details of all welfare matters, including protocol deviations, suspected adverse events, development of concurrent medical problems and euthanasia. In addition, they informed the study observers immediately to agree an appropriate course of action. If withdrawal from the trial proved to be necessary, the study observers recorded the reasons.

For adverse events where the treatment was suspected to be the cause, participation in the study was to be suspended immediately. Where it was thought to be unlikely that an adverse event was related to the treatment, the dog was allowed to continue with the trial, provided that the owners agreed. Participation could also be suspended if an enrolled dog developed an unrelated condition, whilst enrolled in the trial.

Where it became necessary to perform euthanasia (e.g. poor response to therapy, development of another medical disorder), the attending veterinary surgeon would perform this (using overdose of intravenous sodium pentobarbital), after obtaining written consent from the owner.

### Randomisation procedures

#### Sequence generation and allocation concealment

The 40 treatments (20 test and 20 control) were assigned a study number from 1 to 40, based upon a randomised sequence generated in Minitab (Minitab Inc, State College, PA, USA) by the trial statistician (PJC). Treatments were given in sequence according to ascending study number, and each group of four study numbers contained two of each treatment. This was to ensure that numbers remained approximately even throughout the course of the study in case, for whatever reason, it was not possible to recruit all cases. The trial statistician sent the numbered sequence to the treatment manufacturer, who assigned the respective treatments to numbered packaging before posting to the study observers. The treatments were stored away from light and at room temperature, until assigned. The study observers were responsible for allocating dogs to their respective treatment, which occurred in a sequential fashion every time that a new case was enrolled. Neither the study observers nor of any of the attending clinicians were aware of the sequence of treatments.

#### Blinding

A two-stage blinding process was used; the first level ensured that, for the duration of the trial, all owners, attending veterinarians, and study observers were blinded as to what treatment any of the dogs were on. Identical packaging was used for all treatments, consisting of plain plastic pots containing the treatment itself, within plain outer cardboard. The only identifying mark was the study number. As mentioned above, the treatments themselves were identical, comprising granules contained within unmarked gelatin capsules, and organoleptic properties were identical, both before and after the gelatin capsules were opened.

Once all cases had been enrolled, and all dogs had completed the study, the second stage of blinding was then implemented. For this, the trial statistician passed the randomisation sequence to a separate investigator (DB) who was not involved in any other aspect of the trial. This investigator broke the code, randomly assigned a secondary study number to each dog, and assigned the dogs to two groups, named “A” and “B”. A coded spreadsheet of the trial data was then given to the trial statistician, who performed all statistical analyses without knowing which treatment was which. Only when all statistical analyses had been completed were the treatment identities revealed.

### Outcome measures

The primary outcome measure of interest was change in body weight. Secondary outcomes of interest included, change in severity of clinical signs, change in BCS, the dose of treatment used for each dog, and requirement for additional medications. For clinical signs, a composite score was created, by adding together the results of all clinical signs recorded in the questionnaire. All of these outcome measures were decided prior to commencement of the trial.

### Sample size

At the conceptualization stage of the study, the trial statistician (PJC) performed a sample size calculation using a statistical software package (Minitab). The primary outcome measure (percentage gain in body weight) was used and, based upon previous studies [7], the expected mean (± standard deviation) change in body weight was 24 ±15.2%. A 1:1 test:control recruitment rate was assumed and, given that no previous clinical trials had been conducted in canine EPI, a clinically relevant difference in efficacy between treatments of 40% was decided. This figure was based upon the opinions of the study investigators. Calculations assumed that a power of 90% was required to identify this difference with a two-sided *P* of <0.05. Based upon these criteria, it was determined that 20 animals per group would be required.

### Data handling and statistics

Data were entered into an Excel spreadsheet (Microsoft inc.) and checked for errors. Statistical analysis used Minitab 16, STATA12 (Statacorp, College Station, TX, USA), and Stats Direct version 2.6.2 (Stats Direct Ltd., Altrincham, UK). Standard descriptive statistics were used to report baseline data (either median and range, or mean ± standard deviation). Baseline data comparisons were made with Fisher’s exact test (for proportions) or the Mann-Whitney test (for continuous variables). The level of statistical significance was set at *P*<0.05 for 2-sided analyses. Outcome data were analysed both on an intention to treat and per-protocol basis; where there was a discrepancy in results, the former were considered most important. In order to account for missing data in the intention to treat analyses, imputation was performed using the method of “Last Observation Carried-Forward”.

For the primary outcome measure, namely bodyweight, the study design involved repeated measurements of the same animal, and the effect of treatment on weight was, therefore, investigated using a mixed-effects linear regression model in STATA. The *xtmixed* command was used, animal identity was declared as a random effect and estimation was by Maximum Likelihood. The effect of treatment was assessed using a multivariable model, which included treatment group, the visit number and their interaction. Serum cobalamin concentrations were included in the form hypocobalaminaemic and normocobalaminaemic when results were less than and greater than the lower limit of the reference range, respectively. The statistical significance of variables in the model was examined using their Wald statistic or their effect on the deviance.

Enzyme dose and BCS data did not meet the requirements needed for parametric analysis, and were analysed with the signed ranks test (for time differences) and the Mann-Whitney test (for differences between groups at each time-point).

### Protocol changes

A number of required changes were made to study protocol at various stages, mainly because the rate of recruitment of cases was slower than expected. Firstly, the original plan was for all cases to be seen at the SATH; however, initial recruitment was slow and the major hurdle was found to be reluctance to travel. For this reason, compensation for client travel was introduced and administration by the first-opinion veterinarian was then allowed. Second, as based upon the power calculation, the initial intention was to recruit a total of 40 dogs (20 treatment and 20 controls). However, the slow recruitment meant that there were concerns that the treatments would exceed their expiry date, initial set for two years after product manufacture. As a result, two treatments were sacrificed (1 treatment, 1 control) and sent back to the manufacturer so that enzyme activity and microbial contamination could be retested, and enabling an extension to the expiry date to be granted. The first product was one from a dog that had been enrolled, only to withdraw soon after starting (Figure 1). In order to maintain blinding, the trial statistician randomly selected the second product from the remaining treatments, by choosing a treatment opposite to the first sacrificed product.

## Results

### Study centres and dogs

A total of 5 dogs were enrolled at the SATH (control treatment 3, test treatment 2), one practice enrolled 2 dogs (both test treatment), and the remaining 31 practices enrolled 1 dog each. These practices were widely distributed across mainland United Kingdom.

Full details of the timeline of the trial, including withdrawals, are given in Figure 1. Twenty dogs were initially enrolled to the control treatment (uncoated enzyme). Of these dogs, two were withdrawn (and euthanased) within the first 14 days of the trial, one because of a poor response and the other for an unrelated problem (aggression). The enzyme supplied for one of these dogs was ultimately used to confirm product efficacy. A further dog was withdrawn (and euthanased) between days 14 and 28 for a perceived poor response to therapy so that, ultimately, 17 dogs completed the trial. For the test treatment, one enzyme batch was used to confirm product efficacy, meaning that 19 were dogs were enrolled in this group. Of these dogs, one was lost to follow up between days 28 and 56, so that, ultimately, 18 completed the trial.

The baseline characteristics of the two groups are shown in Table 2. No significant group differences for any of the starting characteristics were identified.

**Table 2:** Baseline characteristics of dogs on two different enzyme treatments for canine exocrine pancreatic insufficiency

**Criterion**	**Test treatment (coated)**	**Control treatment (uncoated)**	**P value**
Breed	Border collie (1), CKCS (1),	Akita (2), CKCS (1),	---
	Cocker spaniel (1),	Dogues de Bordeaux (1),	
	GSD (12), Lhasa apso (1), mixed breed (2)	GSD (13), mixed breed (2)	
	West Highland white terrier (1)	Tibetan terrier (1)	
Sex	Male (8)	Male (5)	0.329
	Neutered male (4)	Neutered male (3)	
	Female (2)	Female (7)	
	Neutered female (5)	Neutered female (5)	
Age (months)	41 (12 to 108)	48 (11 to 144)	0.829
Cobalamin^1^	Hypocobalaminemic (5), normocobalaminemic (9),	Hypocobalaminemic (7), normocobalaminemic (9),	0.668
	Not measured (6)	Not measured (4)	

The table reports signalment and cobalamin status in dogs, with exocrine pancreatic insufficiency, randomised to receive one of two enzyme treatments (test e.g. coated and control e.g. uncoated). Numerical data are expressed as median (range). CKCS: Cavalier King Charles Spaniel; GSD: German Shepherd Dog. ^1^Given that various laboratories were used (with different reference ranges), cobalamin data expressed in categories (e.g. normcobalaminaemia, hypocobalaminaemia) rather than as absolute concentrations. There were no differences between groups for any of the baseline parameters.

### Primary outcome measure

**Figure 2:**
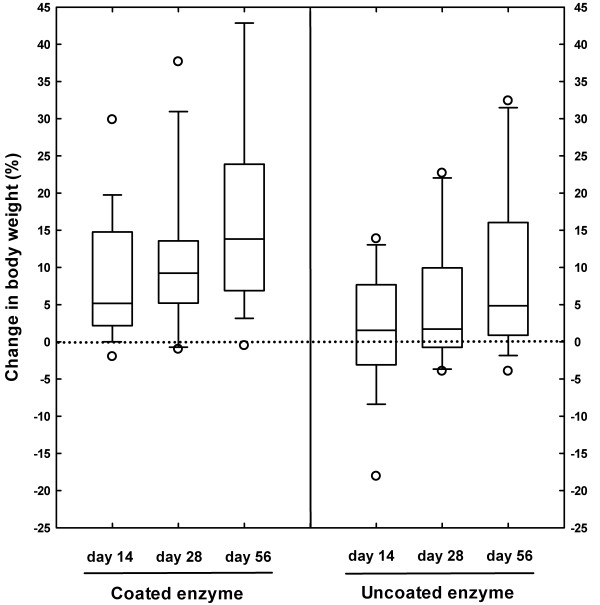
**Effect of pancreatic enzyme replacement on weight gain.** Box and whisker plots illustrating the percentage change in body weight in 40 dogs, with exocrine pancreatic insufficiency, randomised to receive pancreatic enzyme treatment either with or without an enteric coating. The boxes depict median (horizontal line) and inter-quartile range (top and bottom of box), the whiskers show the 10-90% range, and outliers are shown as separate points. Weight gain was significantly greater in the dogs treated with the coated preparation (P<0.01).

Median (range) body weight for both groups is shown in Table 3, and no significant group difference was identified (*P*=0.159). Body weight increased progressively in both groups during the trial (*P*<0.001), and a significant time-group interaction was evident whereby the magnitude of increase was greater for the test treatment than for the control treatment (*P*<0.001; Figure 2). In this respect, by day 56, mean body weight increase was 17% (95% confidence interval 11-23%) in the test treatment group, and increased by a mean of 9% (95% confidence interval 4-15%) in the control treatment group. These findings were similar whether data were assessed on an intention-to-treat (using imputation) or a per-protocol basis (data not shown).

**Table 3:** Outcome variables in dogs on two different enzyme treatments for canine exocrine pancreatic insufficiency

**Criterion**	**Treatment group**		**P value**	
	**Test (coated)**	**Control (uncoated)**	**Time**	**Time-group**
Body weight (kg)				
Before	21 ±8.8 (4 to 36)	26 ±10.3 (6 to 48)	---	0.159
14 days	23 ±9.3 (5 to 40)	26 ±10.1 (7 to 48)	0.962	0.012
28 days	24 ±9.6 (5 to 39)	27 ±10.6 (8 to 48)	0.145	0.004
56 days	25 ±10.0 (6 to 39)	28 ± 10.6 (8 to 48)	<0.001	<0.001
Body condition score^1^				
Before	2 (1 to 5)	2 (1 to 6)	---	0.233
14 days	3 (1 to 5)	2 (1 to 6)	0.018	0.800
28 days	3 (1 to 5)	2 (1 to 6)	<0.001	0.593
56 days	4 (2 to 5)	3 (1 to 6)	<0.001	0.032
Dose of enzyme^2^				
Before	2 (1 to 2)	2 (1 to 4)	---	0.669
14 days	2 (1 to 4)	2 (1 to 7)	0.075	0.311
28 days	2 (1 to 4)	2 (1 to 7)	0.002	0.722
56 days	2 (1 to 4)	4 (1 to 6)	<0.001	0.225
Clinical score^3^				
Before	11 (4 to 19)	13 (2 to 16)	---	0.993
14 days	8 (2 to 12)	7 (2 to 15)	0.003	0.337
28 days	7 (1 to 13)	5 (0 to 11)	0.008	0.476
56 days	7 (1 to 13)	5 (0 to 11)	0.011	0.869
Other therapy				
Before	Oxytetracycline (1)	Cimetidine (1)	---	---
14 days	Oxytetracycline (2)	Oxytetracycline (1)	---	---
28 days	Oxytetracycline (2)	Cimetidine (1) Metronidazole (1) Oxytetracycline (2)	---	---
56 days	Oxytetracycline (1)	Oxytetracycline (3) Ranitidine (1)	---	---

Dogs, with exocrine pancreatic insufficiency, were randomised to receive one of two enzyme treatments (test [e.g. coated] and control [e.g. uncoated]). Numerical data are expressed as median (range), except for body weight, which is expressed as mean ± standard deviation (range). ^1^Body condition score assessed using the 9-point system [5]. ^2^ Dose of enzyme in number of capsules per day. ^3^Clinical score was a composite score for a range of clinical signs, as described in Table 3. Body weight increased progressively in dogs on both treatments (*P*<0.001), but a significant time-group interaction was evident with the magnitude of increase being greater for the test treatment (coated enzyme) than for the control treatment (uncoated enzyme; *P*<0.001). BCS increased over time in both groups (*P*<0.001), but increased more in the test treatment group (*P*=0.032 at 56 days). The dose of enzyme used increased (*P*<0.001 at 56 days) and whilst clinical disease severity score decreased (*P*=0.011 at 56 days) over time, but with no significant treatment group differences noted.

### Secondary outcome measures

The results of all secondary outcome measures, at each visit, are shown in Table 3. As with body weight, BCS increased over time (*P*<0.001 at 56 days). Again, however, there was a group difference, with the BCS of the dogs in the test treatment group increasing more than those in the control treatment group (*P*=0.032 at 56 days). The dose of enzyme used, also increased with time (*P*<0.001 at 56 days), but there was no significant group difference at any time point (*P*=0.225 at day 56). Further, whilst clinical disease severity score decreased significantly over the trial (*P*=0.011 at 56 days), no difference was noted between treatment groups (*P*=0.869 at day 56).

### Ancillary analyses

Given the number of hypocobalaminaemic dogs identified, the group comparison for the primary outcome measure was reassessed, including presence of hypocobalaminaemia as a covariate. The presence of hypocobalaminaemia had a negative effect on weight gain (*P*=0.027). Inclusion of Cobalamin status in the statistical model, of change in body weight, resulted in slightly larger coefficients for the time-group interaction but did not alter the results and conclusions (data not shown).

### Adverse effects

No significant adverse effects were reported, for either treatment, for the duration of the trial.

## Discussion

This study is the first blinded RCT assessing efficacy of pancreatic enzyme therapy for canine EPI, and has been reported in line with the ‘Reporting Guidelines for Randomized Control Trials’ (REFLECT) statement [8]. The principles of evidence-based medicine are now widely accepted in veterinary medicine and, as a result, there is an increasing need to generate objective data to guide clinical decision-making. The findings of this study represent a considerable advance on the prior state of knowledge regarding EPI therapy, which relied on retrospective case series to guide therapy. One such study suggested that enteric-coated products were less effective than uncoated enzyme powders [2], although more recent work suggested that there was no difference in response to therapy between dogs on coated and uncoated products [3]. Whilst these historical results should not be overlooked completely, their findings should be interpreted cautiously, and greater weight given to findings from prospective controlled trials such as the current study.

Based upon the results reported, the study hypothesis that enteric coating of a pancreatic enzyme extract would have no effect on the efficacy of treatment for canine EPI, should be rejected. Not only does this support the use of enteric coated products for treatment of canine EPI, but it emphasises the need to look beyond clinical signs (such as diarrhoea) as evidence of efficacy in this condition. Serial measurement of body weight, using the same set of calibrated electronic weigh scales is a precise means of confirming health, even though it is underused in primary care companion animal practice [9].

Detailed steps were taken to ensure that the order of treatment allocation was concealed from all study investigators and attending veterinarians. In the opinions of the authors, the process of consecutively numbering treatment, according to the predetermined allocation sequence, is superior to other possible approaches e.g. colour-coding the test and control treatments, or labelling products with group identifier (e.g. A or B). Blinding was further maintained by the use of identical packaging and product, so that the products were impossible to identify. A further safety measure came from the fact that the study was small and most cases came from separate practices. As a result, in the unlikely event that an investigator guessed the treatment allocation, this would not be likely to have a major impact on the results of the trial. Finally, in order to ensure that the statistician was also blinded to treatment arms when analysing study data, an additional level of blinding was added subsequent to trial completion. This ensured that, although the subjects comprising each group were known, neither the main study investigators nor the statistician himself could influence the method of analysis. The authors believe that the procedures taken were extremely robust, and suggest that such approaches be considered for companion animal RCTs in the future.

As with any study, limitations must be considered when interpreting findings. Firstly, group sizes were small, and study withdrawals reduced further the ability to make comparisons. In addition to this, trial recruitment was slower than expected, requiring changes in the recruitment protocol (e.g. multi-centre vs. single-centre recruitment) and the sacrifice of two batches of therapy (one test and one treatment) to verify that enzyme activity was maintained. Despite these problems, differences were identified for the primary outcome measure, suggesting that the study power had been sufficient. That said, the use of larger group sizes might have enabled subtle differences in the secondary outcome measures to be identified. The switch from single-centre to multi-centre design was an added complication, and meant that 5 cases were recruited from one centre, with inherent concerns of introducing unwanted influence or possible bias. However, since these 5 cases were, more or less, evenly distributed between treatment groups, it is unlikely that this one centre had any undue influence. The cases from the only other practice to recruit more than one case were randomly assigned to the same treatment group (e.g. test treatment) group. Although not ideal, the effect of this small ‘cluster’ is not likely to have had a major bearing on outcome. A third limitation was the fact that the study was short term and, arguably, a longer period of therapy would have helped to determine whether the treatment advantage identified was maintained, if the relative lack of efficacy of the control treatment could ultimately have been overcome by further dose increases, and if delayed side effects of the therapies developed. That said, initial response (e.g. the first 2-3 months) has been shown to be critical in predicting long-term response [3]. Longer term and larger scale trials are now recommended, to enable a more complete understanding of the treatment advantage that enteric coating provides.

A further study limitation was the fact that diet was not standardised amongst study dogs, and details of exact diets were not recorded as part of the study. Dietary management is recommended as adjunctive therapy in canine EPI, and the most important consideration appears to be in fat content with fat restriction recommended in some [10,11], but not all [12,13], studies. Further, prospective studies have not demonstrated a clear benefit of any specific diet in the treatment of canine EPI [13-15]. In fact, different diet types (i.e. low fat, normal fat high fibre) appeared to suit different dogs, suggesting that such therapy is best tailored to individual response. Therefore, whilst a single diet type for all dogs might have allowed greater uniformity to expected responses, it might actually have confounded the response to therapy, with either a favourable or unfavourable response being the result of the dietary change rather than the enzyme treatments. As a result, clients were advised not to alter diet in anyway, and this would have the benefit that any improvements would then be the result of the enzyme replacement therapy rather than the diet change. Another reason for not switching diets is the fact that many veterinary practices in the UK stock only a limited diet range (e.g. from a single manufacturer), and it would have been difficult to choose a single diet that was acceptable to all participants. Further, altering to a purpose-formulated diet would have cost implications, and might have deterred clients from agreeing to participate. Moreover, there may have been palatability issues with a dietary change, with not all dogs accepting transition to a different diet. Nonetheless, the issue over non-standardised diet is a potential limitation, and future studies regarding EPI therapy should consider standardising the diet between treatment groups.

The demographic of cases recruited was similar to that of previous studies [16], and the majority of cases were seen and managed by primary care veterinarians. This suggests that the results are likely to be relevant and can be generalised to cases of EPI seen in general practice. One limit to generalisability, however, is that, whilst the test treatment was identical to a commercially available product, the control treatment was not. Therefore, the results are relevant to this particular commercial product and also shed light on the principle of using an enteric coating to reduce enzyme degradation, but caution should be exercised when extrapolating to other enzyme preparations. Further, demonstrating that enteric coating is superior in this context does not necessarily prove that the commercial product on which it is based is superior to other commercially available uncoated preparations e.g. uncoated enzyme powder. Although comparing two commercially-available products would arguably have been more relevant for decision-making in clinical practice, this approach would have been more difficult (if not impossible) to blind as thoroughly as was achieved using the test and control treatments in the current study. A further challenge would have been ensuring that such treatments were dosed at a comparative level. Therefore, whilst caution should rightly be exercised when extrapolating the results to all enzyme preparations, the findings are still applicable for guiding therapeutic recommendations in this field.

Hypocobalaminaemia can be seen in the majority of dogs with EPI, and negatively impacts upon long-term survival [3]. More concerningly, despite the laboratory evidence of a deficiency, most EPI dogs with concurrent hypocobalaminaemia do not receive adequate cobalamin supplementation [3]. To reduce the possibility of hypocobalaminaemia being a confounding factor in this trial, one approach would have been to test cobalamin concentrations at each visit, and administer cobalamin injections to dogs that were deficient. However, this would have been costly and might have dissuaded clients and veterinary practices from participating. Since, parenteral cobalamin injections are inexpensive and safe, we decided on the alternative strategy of treating all dogs with weekly cobalamin injections irrespective of their circulating cobalamin concentration. The finding that pre-treatment cobalamin status was negatively associated with treatment response should be interpreted cautiously because it was not a primary aim of the study, and was identified using post-hoc ancillary analyses. Further, since cobalamin status did not differ between groups, and the effect was independent of treatment effect, it was unlikely to have had an effect on the other study findings. Nonetheless, the finding is consistent with the previous regarding concurrent hypocobalaminaemia in EPI patients. Further work is, therefore, required to ascertain the pathological consequences of hypocobalaminaemia in canine EPI, and the impact it may have in response to current therapeutic regimes.

## Conclusions

This study is the first RCT assessing efficacy of enzyme replacement therapy in canine EPI. The study was rigorously blinded, conducted under GCP guidelines and reported according to recommended methods. Based upon the primary outcome measure, dogs receiving an enteric-coated pancreatic enzyme supplement responded better to therapy than those given an otherwise identical uncoated product. Therefore, enteric coating appears to convey a therapeutic advantage for such products, although caution should be exercised when extrapolating these findings to other preparations of pancreatic extract.

## Abbreviations

BCS, Body condition score; EPI, Exocrine pancreatic insufficiency; GCP, Good clinical practice; SATH, Small Animal Teaching Hospital; TLI, Trypsin-like insufficiency.

## Competing interests

VetPlus Ltd., who markets the product on which both treatments were based, funded the trial. However, the sponsors were not involved in study design, enrolment, monitoring or in data analysis. Further, the sponsor could not influence the decision to publish the manuscript.

## Authors’ contributions

AM was the primary study observer responsible for enrolling cases and liaising with primary care veterinarians. AJG and PJN designed the study, helped with case recruitment, acted as study observers, and interpreted the results. PJC was responsible for performing the initial estimation of study size, advising on trial design, conducting the trial randomisation, and analysing the data. DB was responsible for overseeing the second stage of blinding. PG assisted with case recruitment, most notably overseeing the process of advertising to veterinarians submitting canine blood samples found to have low TLI concentration. AJG drafted the first version of the manuscript, which was then edited by all other authors. All authors approved the final version of the manuscript.
